# Relative Importance of Demographic, Socioeconomic and Health Factors on Life Expectancy in Low- and Lower-Middle-Income Countries

**DOI:** 10.2188/jea.JE20130059

**Published:** 2014-03-05

**Authors:** Md. Nazrul Islam Mondal, Mahendran Shitan

**Affiliations:** 1 Laboratory of Computational Statistics and Operations Research, Institute for Mathematical Research, University Putra Malaysia, Selangor Darul Ehsan, Malaysia; 2 Department of Mathematics, Faculty of Science, University Putra Malaysia, Selangor Darul Ehsan, Malaysia; 3 Department of Population Science and Human Resource Development, University of Rajshahi, Rajshahi, Bangladesh

**Keywords:** life expectancy, socioeconomic and health factors, low- and lower-middle-income countries, path analysis

## Abstract

**Background:**

We attempted to identify the pathways by which demographic changes, socioeconomic inequalities, and availability of health factors influence life expectancy in low- and lower-middle-income countries.

**Methods:**

Data for 91 countries were obtained from United Nations agencies. The response variable was life expectancy, and the determinant factors were demographic events (total fertility rate and adolescent fertility rate), socioeconomic status (mean years of schooling and gross national income per capita), and health factors (physician density and human immunodeficiency virus [HIV] prevalence rate). Path analysis was used to determine the direct, indirect, and total effects of these factors on life expectancy.

**Results:**

All determinant factors were significantly correlated with life expectancy. Mean years of schooling, total fertility rate, and HIV prevalence rate had significant direct and indirect effects on life expectancy. The total effect of higher physician density was to increase life expectancy.

**Conclusions:**

We identified several direct and indirect pathways that predict life expectancy. The findings suggest that policies should concentrate on improving reproductive decisions, increasing education, and reducing HIV transmission. In addition, special attention should be paid to the emerging need to increase life expectancy by increasing physician density.

## INTRODUCTION

Life expectancy at birth, a widely used indicator of the overall development of a country, has increased during the last decade in most countries. This indicator is of particular importance in developing countries, as they are striving to achieve socioeconomic progress by substantial investment in social sectors. Life expectancy at birth is defined by the United Nations Human Development Report as “the years a newborn infant would live if prevailing patterns of age-specific mortality rates at the time of birth were to stay the same throughout the child’s life”.

Methods of increasing life expectancy are a primary interest of medical and socioeconomic research. In many parts of the world, life expectancy has been increasing steadily during the past few decades, due to increases in technology, drugs, and international support. People are healthier, wealthier, and living longer today than they were 30 years ago.^1^ Average global life expectancy at birth is predicted to increase by 7 years during the period 1998–2025, and life expectancy at birth will be above 80 years in 26 countries.^1^ Wide variations in life expectancy still exist between high- and low-income countries. Increases in life expectancy have been attributed to improvements in sanitation and access to clean water; medical advances, including childhood vaccines; and massive increases in agricultural production. The level and variability of life expectancy have important implications for individual and aggregate human behavior because they affect fertility behavior, economic growth, human capital investment, intergenerational transfers, and incentives for pension benefit claims.^2^^,^^3^ Life expectancy reflects the health of a country’s people and the quality of care they receive when they are ill.^4^^,^^5^

The demographic and socioeconomic predictors of life expectancy include sex, age, education, and gross national income (GNI) per capita.^4^^–^^7^ Recent studies investigating possible determinants of life expectancy found that the most important determinant was income. Census data from South Korea showed that increased income had a positive impact on life expectancy.^7^ In Thailand, older people with higher incomes and an advanced education had better health outcomes and greater health satisfaction.^8^ Inequalities in income and education were recently found to account for regional inequalities in life expectancy and other health indicators.^9^ Low income and unemployment were found to negatively affect health outcomes.^10^ Significant associations between education and life expectancy were also observed in Brazil,^11^ Finland, Sweden, Norway, Denmark,^12^^,^^13^ and other European countries.^14^ Moreover, longer life expectancy was associated with low infant mortality rates and high literacy rates.^15^

When mortality risks are high, populations typically reproduce frequently (to increase the probability that at least some offspring will survive to maturity) and early (to ensure reproduction before death).^16^ Determinants of life expectancy include healthcare expenditures, healthcare resources, mortality rates, prevalence of human immunodeficiency virus (HIV), and health outcomes.^17^ Healthcare services, such as increasing the numbers of physicians, hospital deliveries, and prenatal examinations, reduce mortality and increase life expectancy.^18^^,^^19^ A number of studies have yielded sound evidence of the effects of demographic events, socioeconomic instability, and availability of healthcare resources on life expectancy.^20^^–^^24^

Research on international inequalities in life expectancy has traditionally focused on overall mortality. However, relatively little research has investigated low- and lower-middle-income countries taken together. The present study hopes to fill this gap in the literature, as it is not only a critical issue in population health research but also a pressing public health concern, with important implications for healthcare policies. Thus, the main purpose of this study was to develop an explanatory model that reveals the relative importance of the factors that contribute to life expectancy. We observed direct, indirect, and total effects of demographic indicators, socioeconomic status, and health factors on life expectancy. We hope that this research will be helpful in identifying factors that directly and indirectly influence life expectancy across countries and that the findings will help policymakers and researchers determine how to optimally allocate their limited resources.

## METHODS

Variables that had the most significant effects on life expectancy in previous studies were selected for study.^7^^,^^20^^–^^24^ Data on low- and lower-middle-income countries were obtained from specialized agencies of the United Nations (UN) system, including the World Health Organization (WHO),^25^ United Nations Development Program (UNDP),^26^ and World Population Data Sheet, Population Reference Bureau.^27^ UN agencies rely on an extensive peer review process, which is conducted through leading regional and national statistics offices and international organizations, thus ensuring the highest level of data consistency and accuracy. The variables investigated in this study, along with their definitions and sources, are shown in eTable 1. A list of the low- and lower-middle-income countries studied is shown in eTable 2. The determinants of life expectancy are grouped into 3 main categories: demographic indicators, socioeconomic status, and health factors. The demographic indicators were total fertility rate (TFR) and adolescent fertility rate (AFR); the socioeconomic variables were mean years of schooling and GNI per capita; and the health factors were HIV prevalence rate and number of physicians per 10 000 population in a given year (physician density).

Data were obtained from 91 low- and lower-middle-income countries. The Solomon Islands, Marshall Islands, and Tuvalu were not included, due to lack of data. Univariate analysis was used to analyze the variables in relation to maximum, minimum, mean, and median values and standard error of the mean (SE mean) and SD. In bivariate analysis, Pearson correlation analysis was used to identify relationships among variables. Finally, path analysis was used to examine the direct, indirect, and total effects of determinants of life expectancy. All statistical analysis was done using STATA software (version 11.0; STATA Corporation, College Station, TX, USA).

Path coefficient analysis is a form of standardized multiple regression analysis and is mainly used to decompose the correlation coefficient, *r*, into direct, indirect, and total effects in order to test the relative importance of each causal effect, as compared with others, on the same dependent variable and to test the conceptual path model for adequacy and parsimony.^28^ The independent variable, *X_i_*, is the *i*-th variable (*i* = 1, 2, 3, … 6), and the dependent variable (*X*_7_) is life expectancy. eTable 1 shows the variables and their measurements used in the path analysis.

On the basis of the causal ordering of variables, the selected set of variables is divided into 2 groups—exogenous variables {X_1_, X_2_, X_3_, X_4_} and endogenous variables {X_5_, X_6_}—and the dependent variable {X_7_}. A main task in this study is to construct a path diagram that visually represents the hypothetical causal relationship between life expectancy and determinants of demographic indicators, socioeconomic status, and health factors. In the recursive path model, each variable is assumed to depend on all prior causal variables.^28^ The system of linear equations for the path model can be written as:

$$\begin{array}{ll} X_{5} & =P_{51}X_{1}+P_{52}X_{2}+P_{53}X_{3}\\ & \;+P_{54}X_{4}+P_{5u}R_{u}; \end{array}$$ (1)

$$\begin{array}{ll} X_{6} & =P_{61}X_{1}+P_{62}X_{2}+P_{63}X_{3}+P_{64}X_{4}\\ & \;+P_{65}X_{5}+P_{6v}R_{v}; \end{array}$$ (2)

$$\begin{array}{ll} X_{7} & =P_{71}X_{1}+P_{72}X_{2}+P_{73}X_{3}+P_{74}X_{4}\\ & \;+P_{75}X_{5}+P_{76}X_{6}+P_{7w}R_{w}; \end{array}$$ (3)

where, *P_ij_* (*i* = 5, 6, 7; *j* = 1, 2, 3, 4, 5, 6) are the path coefficients and *P*_5_*_u_R_u_*, *P*_6_*_v_R_v_*, and *P*_7_*_w_R_w_* are the random disturbance terms.

The path coefficients can be defined as the ratio of the SD of the effect due to a given cause to the total SD of the effect. All random disturbance terms are mutually independent and independent of their corresponding explanatory variables. The residual of the path coefficients can also be estimated from the regression equation as $\sqrt{1-R^{2}}$, where *R*^2^ (unadjusted) is the multiple correlation coefficient (square) of the regression equation for the endogenous variable to which the residual path is attached.^29^ Path analysis reveals the direct, indirect, and total effects of each selected explanatory variable on life expectancy.

## RESULTS

### Univariate analysis

The background statistics for the predictor and response variables for low- and lower-middle-income countries are shown in Table 1, including the maximum and minimum values for all study variables, as well as means, medians, SE means, and SDs. This analysis is useful because the variables are often measured in different units and have very different ranges.

**Table 1.** Descriptive statistics for dependent and independent variables from 91 countries

Variable	No.	Minimum (Country)	Maximum (Country)	Mean	Median	SE Mean	SD
Gross national income (X_1_)	89	265.00 (Liberia)	7694.00 (Korea)	2790.69	2242.00	203.70	1921.69
Mean years of schooling (X_2_)	90	1.20 (Mozambique)	12.10 (Georgia)	5.59	5.25	0.28	2.61
Adolescent fertility rate (X_3_)	90	5.70 (Korea)	207.10 (Niger)	76.39	69.70	5.05	47.88
Total fertility rate (X_4_)	91	1.30 (Korea)	7.10 (Niger)	3.95	3.80	0.15	1.45
Physician density (X_5_)	87	0.10 (Haiti)	45.40 (Georgia)	6.82	2.70	1.03	9.60
HIV prevalence rate (X_6_)	76	0.05 (Korea)	25.90 (Swaziland)	2.56	0.90	0.54	4.72
Life expectancy (X_7_)	91	47.00 (Sierra Leone)	76.00 (Belize)	63.18	65.00	0.93	8.90

Abbreviation: SE, standard error. SD, standard deviation.

Life expectancy at birth was very low among the low-income countries and almost all African countries. Life expectancy was 47 years in Sierra Leone (the lowest value) and 48 years in the Central African Republic, Democratic Republic of Congo, Guinea-Bissau, Lesotho, Swaziland, Zambia, and Zimbabwe. Life expectancy was much longer in developed countries, such as Japan (83 years), Italy (82 years), and Switzerland (82 years).^27^ HIV prevalence rates were highest in low-income countries (Swaziland 25.90%; Lesotho 23.60%; Zimbabwe 14.30%), as compared with a prevalence rate of less than 0.10% in most developed countries.^25^ The number of physicians per 10 000 population was very low in African countries. There was only about 1 physician per 100 000 people in Haiti, Liberia, and Tanzania and 2 physicians per 100 000 people in Niger, Ethiopia, Sierra Leone, Rwanda, and some other countries.^25^ However, TFR and AFR were highest in African countries. TFR was 7.10 in Niger and 6.40 in Somalia as compared with the average of 1.60 in developed countries.^27^ The trend was similar for AFR. In Niger, it was 207.10, which is the highest in the world. In Congo, it was 201.40, the second highest in the world.^26^ Mean years of schooling was very low in low-income countries (Mozambique, 1.20 years; Burkina Faso, 1.30 years).^26^ In addition, GNI per capita was very low in countries with a low life expectancy (eg, GNI per capita in Liberia, US$265).^26^

### Bivariate analysis

Pearson correlation coefficients (*r*) are known as total associations in path analysis and are used to examine the direction, strength, and significance of linear relationships between variables^30^ (Table 2).

**Table 2.** Pearson correlation coefficients between variables

	X_1_	X_2_	X_3_	X_4_	X_5_	X_6_	X_7_
Gross national income (X_1_)	1	0.58^b^	−0.49^b^	−0.68^b^	0.56^b^	−0.20	0.69^b^
Mean years of schooling (X_2_)		1	−0.54^b^	−0.61^b^	0.71^b^	−0.04	0.57^b^
Adolescent fertility rate (X_3_)			1	0.71^b^	−0.48^b^	0.22	−0.64^b^
Total fertility rate (X_4_)				1	−0.62^b^	0.25^a^	−0.76^b^
Physician density (X_5_)					1	−0.28^a^	0.55^b^
HIV prevalence rate (X_6_)						1	−0.55^b^
Life expectancy (X_7_)							1

^a^*P* < 0.05, ^b^*P* < 0.01.

Life expectancy was significantly positively correlated with income, mean years of schooling, and physician density and significantly inversely correlated with AFR, TFR, and HIV prevalence rate. As was the case in univariate analysis, HIV prevalence rate was inversely correlated with physician density, mean years of schooling, and national income and positively correlated with TFR and AFR. Physician density was inversely correlated with TFR and AFR and significantly positively correlated with mean years of schooling and national income. TFR was significantly positively correlated with AFR and significantly inversely correlated with mean years of schooling and national income. Mean years of schooling and national income were significantly inversely correlated with AFR. Mean years of schooling was significantly positively correlated with national income.

### Path analysis

Path coefficients are the direct effects of the determinant factors specified in regression equations (1), (2), and (3) and are estimated by the ordinary least-squares regression procedure. Thus, the fitted form of the illustrative path models is as follows:

$$\begin{array}{ll} & X_{5}=0.14X_{1}+0.50X_{2}+0.03X_{3}-0.26X_{4},\\ & R_{5.1234}^{2}=0.59; \end{array}$$ (4)

$$\begin{array}{ll} & X_{6}=-0.03X_{1}+0.60X_{2}+0.02X_{3}\\ & \;+0.29X_{4}-0.55X_{5},\\ & R_{6.12345}^{2}=0.21; \end{array}$$ (5)

$$\begin{array}{ll} & X_{7}=-0.03X_{1}+0.23X_{2}-0.03X_{3}-0.60X_{4}\\ & \;-0.11X_{5}-0.42X_{6},\\ & R_{7.123456}^{2}=0.81. \end{array}$$ (6)

Fundamental to path analysis is the path diagram, which is the outcome of a set of linearly interrelated variables and the assumed causal relationships among them. In path analysis, the variables involved in the path diagram are divided into 3 groups—the exogenous variables, endogenous variables, and residual variable. These 3 types of variables are diagrammatically presented in the Figure, which is the fitted form of the above path models.

**Figure.**
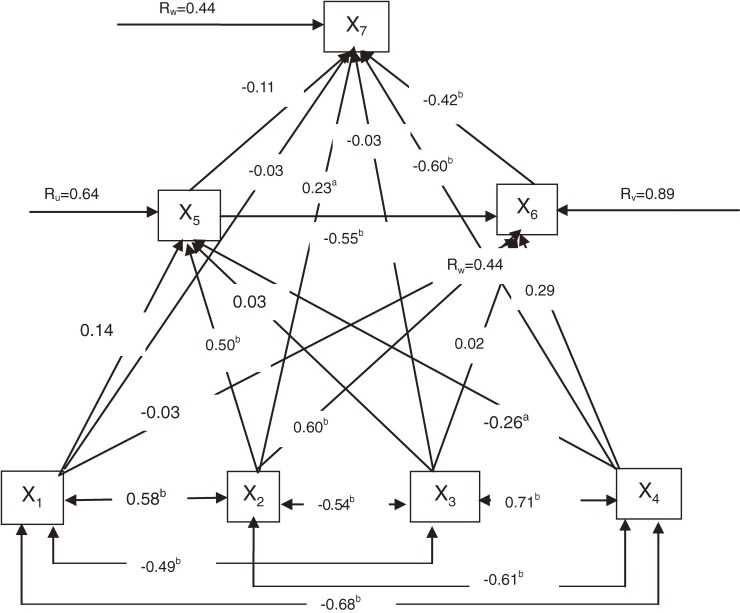
Path diagram of factors affecting life expectancy. ^a^*P* < 0.05, ^b^*P* < 0.01.

The direct, indirect, and total effects and various path coefficients of the explanatory variables from path analysis were obtained, and the interpretations of the effects of these factors on life expectancy are shown in Table 3.

**Table 3.** Effects of demographic, socioeconomic, and health determinants on life expectancy

Endogenous variable	Exogenous variable	Total effect	Non-causal effect	Indirect effect via		Direct effect	Total association

				X_5_	X_6_		
X_5_	X_1_	0.14	−0.42	—		0.14	0.56^b^
	X_2_	0.50	−0.20	—		0.50^b^	0.71^b^
	X_3_	0.03	0.51	—		0.03	−0.48^b^
	X_4_	−0.26	0.36	—		−0.26^a^	−0.62^b^

X_6_	X_1_	−0.10	0.10	−0.08		−0.03	−0.20
	X_2_	0.32	0.36	−0.28		0.60^b^	−0.04
	X_3_	0.01	−0.21	−0.01		0.02	0.22
	X_4_	0.43	0.18	0.14		0.29	0.25^a^
	X_5_	−0.55	−0.27	—		−0.55^b^	−0.28^a^

X_7_	X_1_	−0.07	−0.76	−0.06	0.01	−0.03	0.69^b^
	X_2_	−0.23	−0.80	−0.21	−0.25	0.23^a^	0.57^b^
	X_3_	−0.05	0.59	−0.01	−0.01	−0.03	−0.64^b^
	X_4_	−0.61	0.15	0.11	−0.12	−0.60^b^	−0.76^b^
	X_5_	0.12	−0.43	—	0.23	−0.11	0.55^b^
	X_6_	−0.42	0.13	—	—	−0.42^b^	−0.55^b^

X_1_ = gross national income, X_2_ = mean years of schooling, X_3_ = adolescent fertility rate, X_4_ = total fertility rate, X_5_ = physician density, X_6_ = HIV prevalence rate, X_7_ = life expectancy.

Non-causal effect = Total effect − Total association.

Total effect = Direct effect + Indirect effect.

^a^*P* < 0.05, ^b^*P* < 0.01.

Table 3 shows the total associations among variables, ie, the direct effects, non-causal effects, and total effects of exogenous and endogenous variables on the residual variable (life expectancy). Table 3 also shows the direct effects of exogenous variables, through endogenous variables, on the residual variable. The total association with life expectancy was statistically significant for all predictors. In addition, the direct effects of mean years of schooling, TFR, and HIV prevalence rate on life expectancy were statistically significant (Model 6). Moreover, the direct effects of physician density and mean years of schooling on HIV prevalence rate were statistically significant (Model 5), as were the direct effects of TFR and mean years of schooling on physician density (Model 4). GNI and physician density had favorable indirect effects, and mean years of schooling, AFR, and TFR had adverse indirect effects, on life expectancy through HIV prevalence rate. TFR had a favorable indirect effect, and GNI, mean years of schooling, and AFR had adverse indirect effects, on HIV prevalence rate through physician density. Similarly, TFR had a favorable indirect effect, and GNI, mean years of schooling, and AFR had adverse indirect effects, on life expectancy through physician density. Finally, among determinant factors, physician density had a favorable total effect on life expectancy in low- and lower-middle-income countries.

## DISCUSSION

Bivariate analysis showed that life expectancy was significantly associated with GNI, mean years of schooling, AFR, TFR, physician density, and HIV prevalence rate. Path analysis revealed that HIV prevalence rate, TFR, and mean years of schooling were significant direct predictors of life expectancy in low- and lower-middle-income countries. These findings are important because they show that health is linked with policy and economics at the country level and because they highlight the direction of health policy worldwide.

Economic development determines improvements in social conditions and increases life expectancy. The residents of a country with high living standards live longer, on average, and have a lower mortality rate.^8^ One measure of a country’s standard of living is per capita GNI, which this study consistently showed was strongly related to life expectancy. There is considerable evidence linking income inequality to poor health outcomes. Low- and lower-middle-income countries obviously have less to spend on preventive medicine and healthcare, which might explain why average longevity is much shorter in such countries. We also found a statistically significant relationship between income distribution and life expectancy in this study, which confirms previous findings on the effects of GNI on average life expectancy. Our results show that higher income increases life expectancy, in accordance with the findings of previous studies.^10^^,^^23^ This finding has important implications: in addition to implementing economic reconstruction programs, such as increasing job opportunities, policymakers should be made aware that economic hardship can affect vulnerable populations such as elderly people, whose health status might deteriorate. A previous study found that death rates among elderly adults were substantially higher in lower-income groups.^8^ Economic upturns are associated with greater life expectancy rates, and the opposite is true for economic downturns.

Education is another influential factor in life expectancy and has direct and indirect effects on health outcomes. We found that higher education levels among a population had a positive impact on life expectancy: the correlation coefficient for mean years of schooling was statistically significant and positive. This finding has important implications, ie, that higher education levels are associated with more timely receipt of healthcare and greater health awareness. People with more education are likely to be better aware of the need to obtain adequate prenatal care and can be encouraged to optimize the use of maternal health services, thereby avoiding childbirth-related complications such as low birth weight. Individuals with more education typically earn higher real wages, which means that average household income is higher, enabling people to increase the quality and quantity of the healthcare services they purchase. Moreover, people with more education tend to better understand information on proper nutrition, hygiene, healthcare services, and common illness prevention measures. In the last decade, the education sector has had a progressively more important role in HIV prevention. Even in the worst-affected countries, school-age children have the lowest HIV infection rates. Providing young people, especially girls, with the “social vaccine” of education offers them a real chance for a productive life. For example, the education sector began an accelerated initiative program in 2002–2006 in 37 sub-Saharan African countries with a total of 200.2 million school-age children and 2.6 million teachers. The efforts to date have the potential to benefit 85.50% of school-age children and 74.30% of primary and secondary school teachers in the region.^31^ Thus, we conclude that average life expectancy will increase as average years of schooling increases.

High fertility may also have a negative effect on life expectancy, as high-fertility families have limited resources per child and because a short period between births may decease breast-feeding and endanger the nutritional status of infants. In the present study, TFR and AFR were significantly inversely correlated with income and education. In addition, life expectancy was inversely correlated with AFR and TFR. TFR had a significant direct effect on life expectancy. Importantly, adolescents all over the world are exposed to excess reproductive-health hazards, as more than 14 million adolescents aged 15 to 19 years give birth each year.^32^ Adolescent fertility is associated with adverse maternal and child health outcomes, including obstructed labor, low birth weight, fetal growth retardation, and high infant and maternal mortality.^33^^,^^34^ Greater adolescent fertility leads to higher prevalence of sexual activity among unmarried girls and exposes them to unplanned pregnancies, unsafe abortions, and sexually transmitted diseases, including HIV.^35^ Delayed reproduction increases survivorship, a relationship that has been observed worldwide.^36^ Studies have reported that adolescents who anticipate a shorter lifespan reproduce at an earlier age than adolescents who expect to live longer.^37^^–^^39^

The causal links between life expectancy and reproduction depend on the stage of demographic transition that a population is experiencing. Countries vary in the rate at which they pass through the stages of demographic transition. Some countries, such as China, Brazil, and Thailand, have moved rapidly through these stages, as a result of economic and social changes. Other countries, particularly those in Africa, have stalled owing to economic stagnation and the impact of HIV infection.^40^ Therefore, the relationships between life expectancy and reproduction depend on economic factors and disease indicators. Indeed, these factors may account for the lack of a correlation between life expectancy and age at first birth among populations with low life expectancies (<60 years).

Physician density was significantly positively correlated with income and education but significantly inversely correlated with TFR and AFR. If there are too few medical personnel to treat the general population, most individuals would likely not receive ordinary medical care. Screening is the best way to identify early HIV infection and is a routine component of primary care, and physician care is involved in monitoring people with HIV infection. Diagnosis can occur at any stage of HIV infection. Appropriate management with combination antiretroviral therapy can extend life, sometimes for many years.^41^ Thus, it is hypothesized that average life expectancy will increase as the number of physicians per 10 000 people increases. Availability of and access to healthcare services is an important factor in protecting against disease onset and accelerating recovery from illness and disability. We found that physician density was significantly positively associated with life expectancy. This is consistent with most previous research in Western countries, which highlights the important role that healthcare access has in the survival of children and older people.^9^^,^^19^ It has been shown that greater healthcare availability in rural areas increases the survival and health of older people.^17^ In addition, maternal and fetal/neonatal survival depends on a continuum of basic services through pregnancy, delivery, and the neonatal period.^4^ However, inadequate access to healthcare services for severe childhood illness could affect psychological development and accelerate declines in organ function during adulthood. These obstacles might reduce an individual’s reserve capacity to resist disease, thus increasing mortality and health problems at later ages, leading to reduced life expectancy. This might explain our findings regarding the association of physician density and life expectancy.

HIV can lead to incurable disease that eventually attacks the immune system of infected individuals. Without treatment, net median survival time with HIV is 9 to 11 years, meaning that people with HIV have a much shorter life span. The existence of a greater percentage of infected adults could also mean higher rates of HIV transmission to children. Thus, it is unsurprising that an increased HIV prevalence rate corresponds to decreased life expectancy. Regional differences are also present, as many countries with high HIV prevalence rates experience a drop in life expectancy.^42^ The results of the rapid and marked increase of HIV on life expectancy were seen in the relationship between life expectancy and HIV prevalence rate in low-income countries.^43^ Importantly, the last decade of the 20th century was a period of stagnation, and inequalities in overall life expectancy increased largely because of the decline in life expectancy in sub-Saharan Africa, which was caused by the HIV epidemic. The present study included 91 countries, 44 of which were low- and lower-middle-income African countries. Deaths will occur in a country or region when the health inventory decreases to a certain level.^24^

The large gap in life expectancy between low-/lower-middle-income countries and developed countries is caused by demographic, socioeconomic, and health factors. However, the adverse effects of these factors may contribute to unequal access and control over maternal and non-maternal resources, unfair division of labor, inequalities in early childhood development, and barriers to educational attainment that discriminate against women. As a result, demographic and socioeconomic mechanisms that lead to unequal distribution of health-enhancing factors are increasingly important obstacles to gains in life expectancy. Thus, increases in AFR, TFR, and HIV prevalence are likely to decrease average life expectancy in a country. In contrast, increased income and education are likely to increase life expectancy.

A limitation of this study is that we only analyzed data for the most common determining factors, ie, those that were found to be significantly associated with life expectancy in previous studies. In addition, the analysis was limited to low- and lower-middle-income countries. The present study identified pathways and determinants of life expectancy, but did not investigate the determinants that explain sex differences in life expectancy in these countries. Data on the 91 counties were obtained from specialized UN agencies. However, the sources and quality of data vary according to country. Some low-income countries have comprehensive civil registration and vital statistics and regular censuses of the entire population. However, many lower-middle-income countries have incomplete or dysfunctional birth and death registration systems and therefore lack continuous empirical data on mortality and life expectancy.

## CONCLUSION

We analyzed how demographic events, socioeconomic status, and health factors affect life expectancy in low- and lower-middle-income countries and clearly identified factors that could improve average life expectancy in those countries. Strong interactions were found among life expectancy, income, educational attainment, fertility, health facilities, and HIV prevalence. Greater education, physician density, and national income were associated with significantly higher average life expectancy. In contrast, demographic changes and health factors were more likely to increase life expectancy by decreasing AFR, TFR, and HIV prevalence. Our analysis also identified the direct, indirect, and total effects of determinant factors on life expectancy.

Our results have policy implications for these countries, especially those in Africa. Urgent action is necessary to enhance life expectancy. International efforts should aim to increase average life expectancy by increasing the number of physicians in these countries. Targeted efforts should empower adolescents to resist early cohabitation, promote education, and encourage family planning. We analyzed data from 91 countries and measured the effects of 6 demographic, socioeconomic, and health determinants. To identify the factors that influence average life expectancy, future research should evaluate larger datasets and a wider range of factors.

## ONLINE ONLY MATERIALS

eTable 1. Descriptions and sources of study variables.

eTable 2. Countries analyzed, by geographic region^a^ (*N* = 91).
