# Supplementary material for: Relative Importance of Demographic, Socioeconomic and Health Factors on Life Expectancy in Low- and Lower-Middle-Income Countries
Source: J Epidemiol. 2014 Mar 5;24(2):117–24. doi: 10.2188/jea.JE20130059 (PMC3956691; doi:10.2188/jea.JE20130059)
Supplement: eTable 1. [file je-24-117-s001.pdf]

eTable 1. Descriptions and sources of study variables

Variable	Description	Source
Gross national income (GNI) per capita (X_1)	GNI PPP per capita is gross national income in purchasing power parity (PPP) divided by mid-year population. GNI PPP refers to gross national income converted to "international" dollars using a purchasing power parity conversion factor. International dollars is the amount of goods and services one could buy in the United States with a given amount of money.	26
Mean years of schooling (X_2)	Average number of years of education among adults 25 years or older, converted from educational attainment levels (using the official durations of each level)	26
Adolescent fertility rate (X_3)	Number of births to women aged 15-19 years per 1000 women aged 15-19 years	26
Total fertility rate (TFR) (X_4)	Average number of children born to a woman, assuming that current age-specific birth rates remained constant throughout her childbearing years (age 15-49 years)	27
Physician density (X_5)	Number of physicians per 10,000 population	25
HIV prevalence rate (X_6)	Estimated number of HIV-positive adults aged 15-49 years per 100 population	25
Life expectancy (X_7)	Average number of years a newborn infant can expect to live under current mortality levels	27
