# Supplementary material for: Relative Importance of Demographic, Socioeconomic and Health Factors on Life Expectancy in Low- and Lower-Middle-Income Countries
Source: J Epidemiol. 2014 Mar 5;24(2):117–24. doi: 10.2188/jea.JE20130059 (PMC3956691; doi:10.2188/jea.JE20130059)
Supplement: eTable 2. [file je-24-117-s002.pdf]

eTable 2. Countries analyzed, by geographic region[a] (N=91)

Region	No.	Country	
		Low income	Lower middle income
**Africa**	**44**		
Eastern Africa	15	Burundi, Comoros, Eritrea, Ethiopia, Kenya, Madagascar, Malawi, Mozambique, Rwanda, Somalia, Uganda, United Republic of Tanzania, Zambia, Zimbabwe	Djibouti
Central Africa	7	Central African Republic, Chad, Democratic Republic of the Congo	Angola, Cameroon, Congo, Sao Tome and Principe
Northern Africa	4		Egypt, Morocco, Sudan, Tunisia
Southern Africa	2		Lesotho, Swaziland
Western Africa	16	Benin, Burkina Faso, Gambia, Ghana, Guinea, Guinea-Bissau, Liberia, Mali, Mauritania, Niger, Sierra Leone, Togo	Cape Verde, Côte d'Ivoire, Nigeria, Senegal
**Oceania**	**6**		Kiribati, Micronesia, Papua New Guinea, Samoa, Tonga, Vanuatu
**Asia**	**26**		
Eastern Asia	4	Democratic People's Republic of Korea	China, Maldives, Mongolia
Southern Asia	7	Bangladesh, Myanmar, Nepal	Bhutan, India, Pakistan, Sri Lanka
Southeast Asia	6	Cambodia, Lao People's Republic	Indonesia, Philippines, Thailand, Vietnam
Western Asia	9	Afghanistan, Kyrgyzstan	Armenia, Georgia, Iraq, Jordan, Syrian Arab Republic, Timor-Leste, Yemen
**Latin America and the Caribbean**	**10**		
Caribbean	1	Haiti	
Central America	5		Belize, El Salvador, Guatemala, Honduras, Nicaragua
South America	4		Bolivia, Ecuador, Guyana, Paraguay
**Southern and Eastern Europe**	**0**		
Eastern Europe	0		
Southern Europe	0		
**Eurasia**	**5**	Tajikistan	Republic of Moldova, Turkmenistan, Ukraine, Uzbekistan

[a]Based on the geographic regions specified by the United Nations
